# Abdominal Pain, Distension, and Dysuria in a Patient with a Solitary Kidney

**DOI:** 10.34067/KID.0000000000000236

**Published:** 2023-12-28

**Authors:** Allina P. Flores-Mendoza, Víctor F. Camacho-Trejo, Lilia M. Rizo-Topete

**Affiliations:** 1 Hospital Universitario Dr. Jose Eleuterio Gonzalez, Monterrey, Mexico; 2 Instituto Mexicano Del Seguro Social, Monterrey, Mexico

**Keywords:** AKI, albuminuria, CKD, kidney anatomy, kidney stones

## Abstract

**Figure:**
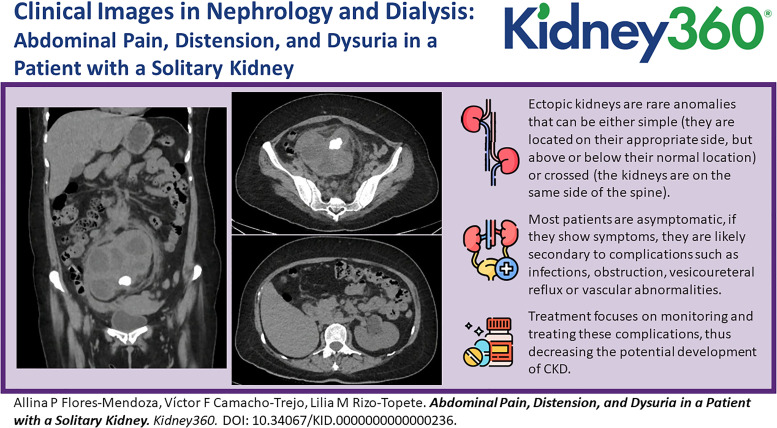

## Case Description

A 39-year-old woman known to have a solitary kidney presented with a 2-week history of abdominal pain localized to the hypogastric region, accompanied by abdominal distension and dysuria, but no fever. On admission, she was afebrile (36°C), BP was at her baseline of 90/70 mm Hg, heart rate was 110 beats/min, and respiratory rate was 12 breaths/min. Physical examination revealed tenderness in the umbilical and hypogastric regions, but not at the costovertebral angles. Pertinent blood tests showed a white blood cell count of 10,840/*µ*l and hemoglobin of 10.3 g/dl (hypochromic microcytic anemia). Her kidney function was normal (creatinine and BUN were 0.6 and 6.4 mg/dl, respectively). The urinalysis was characterized by microscopic hematuria (5 erythrocytes/high power field), pyuria (100 leukocytes/high power field), and low-grade proteinuria (100 mg/dl). A computerized tomography urogram was performed.

**Figure 1:**
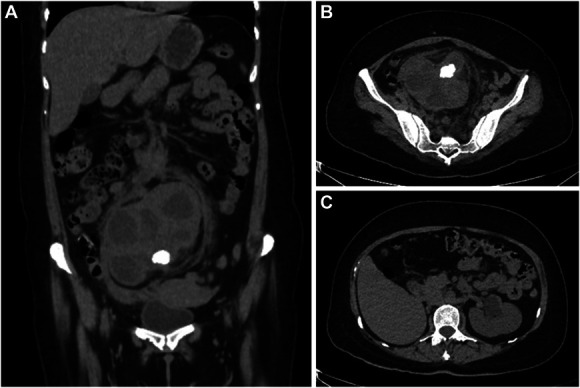
**CT Urogram images.** (A) Coronal plane of the CT urogram. The ectopic kidney is seen in the lower abdomen. It is enlarged because of hydronephrosis, caused by the obstructing renal stone seen at the hilum. (B) Transverse plane of CT urogram (lower abdomen). The hydronephrotic ectopic kidney is again seen, together with the kidney stone in its pelvis and perirenal fatty striation. (C) Transverse plane of the CT urogram. The left kidney is located in a normal position. The presence of mild–moderate hydronephrosis with mild ureteral ectasia is also evident. CT, computerized tomography.

The computerized tomography urogram (Figure 1, A–C) revealed the presence of two kidneys, not a solitary one as previously reported. However, the right kidney was ectopic, in that it was in the pelvic region. It had well-defined, albeit lobulated borders and was enlarged because of hydronephrosis, measuring 142×94×106 mm. There was increased perirenal fatty striation and a well-demarcated hyperintensity (27 mm in size) indicating a kidney stone (Figure 1, A and B). The left kidney was in its normal position and had normal dimensions (137×67×69 mm), except that the kidney hilum was dilated (22 mm), suggesting mild-to-moderate hydronephrosis (Figure 1C). The patient was treated with antibiotics for 2 days at which time she underwent a percutaneous nephrolithotomy. She completed her course of antibiotics and had a complete resolution of her symptoms.

## Discussion

Ectopic kidneys are rare congenital anomalies, characterized by the abnormal location of one or both kidneys. They are divided into two types. Simple ectopia refers to when the kidneys are on the appropriate side of the spine, but one or both will be above or below their normal location. Crossed ectopia is when both kidneys are located on the same side of the spine.^1^ The incidence of ectopic kidneys varies on the basis of location and type of ectopy, the most common being one normal and one ectopic pelvic kidney (1 in 3000). Other types include crossed kidney ectopy (1 in 7000), ectopic thoracic kidney (1 in 13,000), and solitary pelvic kidney (1 in 22,000).^2^ Kidney ectopy may also be associated with other malformations of the genitourinary tract. The presence of an ectopic kidney is most frequently an incidental discovery in imaging studies being performed for unrelated concerns. While most patients are asymptomatic, the ones who experience symptoms are usually due to the complications arising from the ectopic location, such as urinary tract infections, stones, and obstruction.^3^ The symptoms in these patients may be classic (*e.g.*, abdominal pain, hematuria, dysuria), but their location is often atypical because of the abnormal location of the kidney. The ectopic location may also render these kidneys more prone to other complications, such as traumatic injury, hypertension (due to kidney vascular abnormalities), and chronic kidney injury if vesicoureteral reflux or obstruction is present. Management of these individuals is limited to periodic monitoring for potential complications of the ectopy (*e.g.*, infections, obstruction, hypertension, CKD) and treating them as needed. In rare cases of patients with frequent and/or severe complications, a more aggressive approach (*e.g.*, nephrectomy) may be warranted.

## Teaching Points

- Ectopic kidneys are rare anomalies that can be either simple (they are located on their appropriate side, but above or below their normal location) or crossed (the kidneys are on the same side of the spine).
- Most patients are asymptomatic; if they show symptoms, they are likely secondary to complications, such as infections, obstruction, vesicoureteral reflux, or vascular abnormalities.
- Treatment focuses on monitoring and treating these complications, thus decreasing the potential development of CKD.
